# Outbreak Trends of Coronavirus Disease–2019 in India: A Prediction

**DOI:** 10.1017/dmp.2020.115

**Published:** 2020-04-22

**Authors:** Sunita Tiwari, Sushil Kumar, Kalpna Guleria

**Affiliations:** Department of Computer Science, Govind Ballabh Pant Engineering College, Delhi, India; Chitkara University Institute of Engineering and Technology, Chitkara University, Punjab, India

**Keywords:** COVID-19 epidemic trend, COVID-19 outbreak in India, predictive model, machine learning

## Abstract

**Objective:**

The objective of this paper is to prepare the government and citizens of India to take or implement the control measures proactively to reduce the impact of coronavirus disease 2019 (COVID-19).

**Method:**

In this work, the COVID-19 outbreak in India has been predicted based on the pattern of China using a machine learning approach. The model is built to predict the number of confirmed cases, recovered cases, and death cases based on the data available between January 22, 2020, and April 3, 2020. The time series forecasting method is used for prediction models.

**Results:**

The COVID-19 effects are predicted to be at peak between the third and fourth weeks of April 2020 in India. This outbreak is predicted to be controlled around the end of May 2020. The total number of predicted confirmed cases of COVID-19 might reach around 68 978, and the number of deaths due to COVID-19 are predicted to be 1557 around April 25, 2020, in India. If this outbreak is not controlled by the end of May 2020, then India will face a severe shortage of hospitals, and it will make this outbreak even worse.

**Conclusion:**

The COVID-19 pandemic may be controlled if the Government of India takes proactive steps to aggressively implement a lockdown in the country and extend it further. This presented epidemiological model is an effort to predict the future forecast of COVID-19 spread, based on the present scenario, so that the government can frame policy decisions, and necessary actions can be initiated.

The World Health Organization (WHO) office of China received information on the first case of pneumonia of unknown etiology on December 31, 2019, in Wuhan city of China.^1^ In the next 4 days (December 31, 2019 – January 3, 2020), another 44 cases of similar type were reported in China; however, the reason was still unidentified. Further, on January 7, 2020, China declared that they had identified an advanced type of coronavirus. The virus is called *severe acute respiratory syndrome coronavirus–2 (SARS-CoV-2)*, and the WHO named the disease, *coronavirus disease 2019 (COVID-19)*.

COVID-19 originated in China; however, since the last 3 months, it has become a public health threat and has spread almost all over the world.^2^ On March 11, 2020, the WHO declared the virus outbreak a pandemic. As of April 3, 2020, the total number of confirmed cases reached 1 026 688 in 199 countries and territories, which has resulted in 54 045 deaths.^2^ There is no vaccine or specific drug combination available to control the effect of COVID-19.^3^ Therefore, to control the loss of lives, governments of most of the countries are imposing several restrictions on their citizens.^4^

The first confirmed case of COVID-19 in India was reported on January 30, 2020, and on April 3, 2020, a total of 2567 cases were confirmed. It took 15 days for the number of novel coronavirus cases in India to go from 100 to 1000, whereas, in Turkey, it took only 4 days. The Indian Government imposed a complete lockdown for 21 days, that started from March 25, 2020, to April 14, 2020, to control the outbreak. It is urgent to study the COVID-19 cases of China to prepare a situation report of India for the coming days so that the government, authorities, and citizens can take or implement the control measures proactively.

This study is focused on predicting the outbreak trends in India based on the pattern of the outbreak in China, considering that the population density of both countries is very high. The prediction model is built from the publicly available dataset of COVID-19. This dataset includes the daily numbers of cumulative confirmed cases, recovered cases, and death cases from January 22, 2020, to April 3, 2020. This prediction model predicts the daily number of cumulative confirmed cases, recovered cases, and death cases from April 4, 2020, to April 25, 2020.

## METHODS

This section covers the details of methods used for predicting COVID-19 cases in India.

### Dataset and Methodology

**FIGURE 1:**
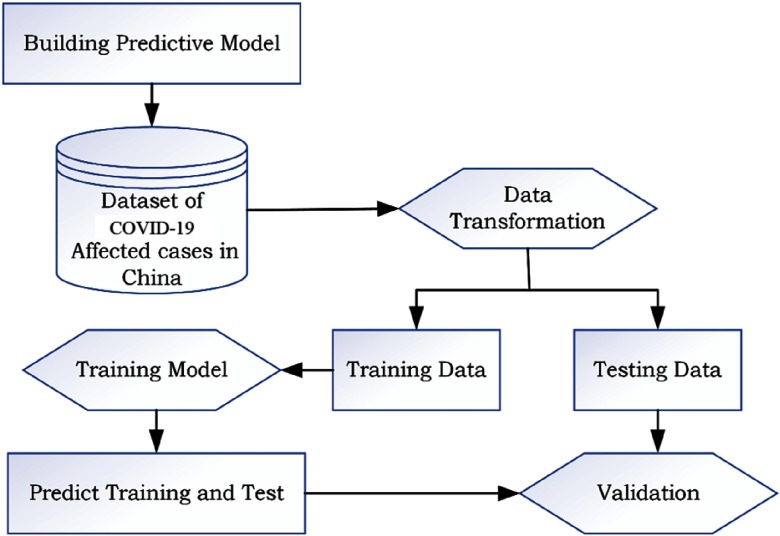
Model Overview.

The dataset of COVID-19 has been downloaded from Kaggle. The dataset is contributed by the Center for Systems Science and Engineering (CSSE) at Johns Hopkins University (JHU).^5^ This dataset includes nationwide details of confirmed cases, recovered cases, and death cases. The presented work uses the data of China from January 22, 2020, to April 3, 2020, to predict the outcome in India in the next 22 days. A predictive model is built using WEKA to predict the day-wise number of confirmed cases, recovered cases, and death cases.^6^ Time series forecasting is performed on the data collected, and a model is prepared. The daily numbers of confirmed cases and their corresponding days are given as input to obtain the relationship between both variables. The predictive models are derived from the data available from China on the assumption that the COVID-19 epidemic trend in India is similar to that in China with a time lag. This assumption is based on the high population density of both countries. Figure 1 shows the model overview. Each stage is detailed with the following information.

#### Dataset of COVID-19 Cases in China

Data of confirmed cases by date, recovered cases, and death cases of China are filtered from the dataset. Additionally, the available data for India are also filtered for validation.

#### Data Transformation

Data are transformed for processing and stored in.csv files for further processing.

#### Training and Test Data

The database of China is divided into training data and test data; 70% of the data is used for training the predictive model, and the remaining 30% is used for testing.

#### Training Model

The aim of the training model is to fit the model using the training data. The model is trained using the time series forecasting methods. After training the model, prediction models are tested to evaluate the performance in testing datasets.

#### Validation

In the validation step, the accuracy of results is verified, comparing the test data with predictions.

## RESULTS

**FIGURE 2:**
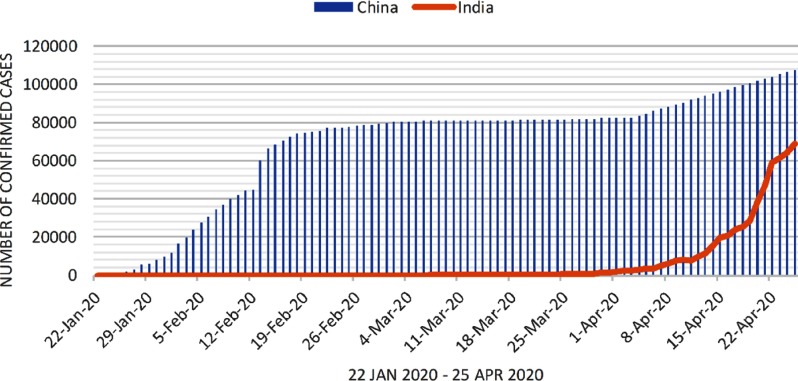
Number of Confirmed Cases in India From January 22, 2020, to April 25, 2020.

The predictive model is built for predicting the daily number of confirmed cases of COVID-19 in India. We obtained the predictive model to predict the number of confirmed COVID-19 cases. The confirmed cases in India and China are shown from January 22, 2020, to April 25, 2020, in Figure 2. From this figure, it is concluded that the number of cumulative confirmed cases in India is likely to increase at a swift pace after April 6, 2020. As per this prediction model, India may have nearly a million confirmed cases by the end of May 2020. This may be controlled if the climatic conditions and Government of India policies become favorable to control the virus.

**FIGURE 3:**
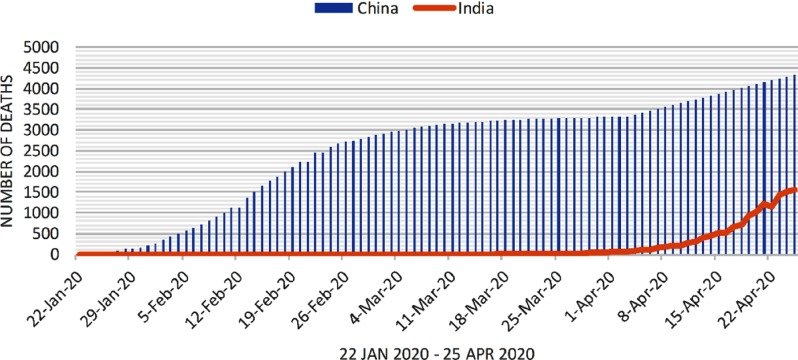
Number of Death Cases in India From January 22, 2020, to April 25, 2020.

Similarly, the predictive model is built for predicting the daily number of COVID-19 deaths due to the pandemic in India. The results for this are shown in Figure 3. The number of death cases from COVID-19 is predicted to increase around April 5, 2020. Around April 25, 2020, the number of death cases in India is expected to be around 1557. This number may further increase after April 25, 2020. The overall mortality rate in India is expected to be lower as compared to the rest of the world. The demographic characteristics of India differ from other countries. In 2020, approximately 8.5% of the Indian population are age 60 years and older, whereas this number is more than 23% in Italy.^7-8^ COVID-19 is fatal in older patients, and therefore the fatality rate of India is lower as compared with that of other countries as of April 3, 2020.

**FIGURE 4:**
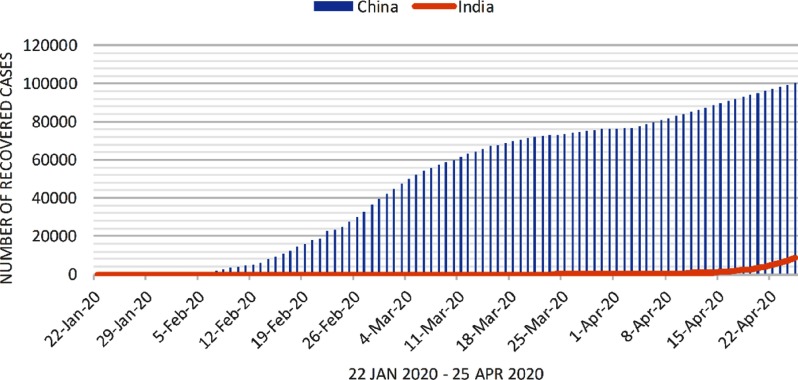
Number of Recovered Cases in India From January 22, 2020, to April 25, 2020.

Along the same lines as above, the model is built for predicting the daily number of recovered cases of COVID-19 in India, as shown in Figure 4. This number is expected to increase after April 25, 2020. As discussed previously, by the end of May 2020, India may have around 1 million confirmed cases, and there could be a severe shortage of hospital beds by June 2020. This may lead to an increase in case fatality.

**FIGURE 5:**
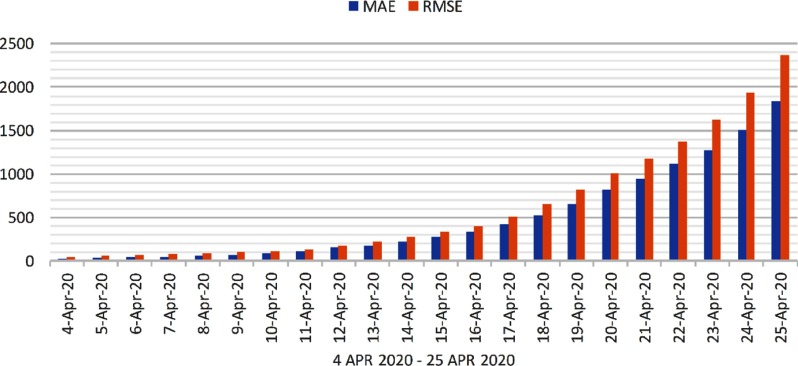
Evaluation of Confirmed Cases From April 4, 2020, to April 25, 2020.

**FIGURE 6:**
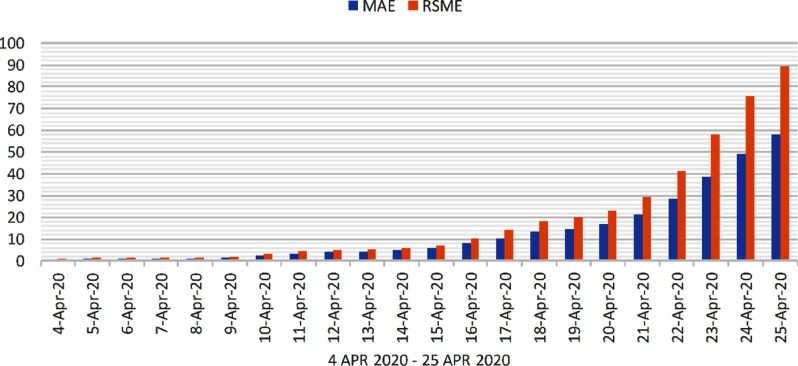
Evaluation of Death Cases From April 4, 2020, to April 25, 2020.

**FIGURE 7:**
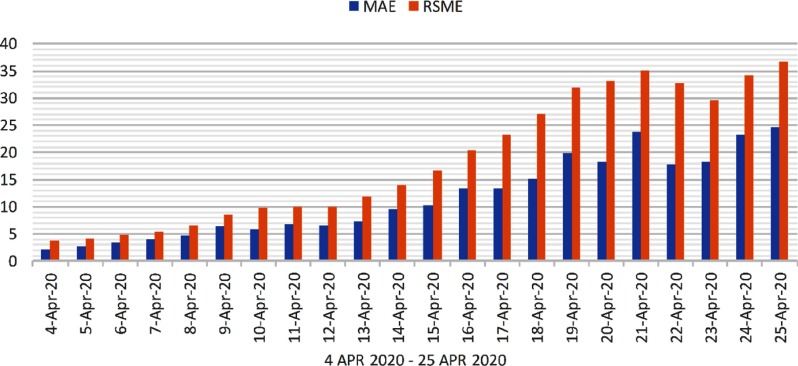
Evaluation of Recovered Cases From April 4, 2020, to April 25, 2020.

Evaluation is performed on confirmed cases, death cases, and recovered cases. The 2 popular metrics for evaluation are mean absolute error (MAE) and root mean square error (RMSE). The MAE and RMSE values of predicted 22 days are shown in Figures 5, 6, and 7, respectively, for confirmed cases, recovered cases, and death cases in India.

## DISCUSSION

The eruption of the novel coronavirus COVID-19 had left extensive and profound impacts worldwide. Although this disease appears to be well controlled in India till now, the recent dramatic increase in new cases and deaths outside of India, especially in Europe and the United States, indicates that the COVID-19 eruption may have tragic results globally if mitigation measures are not implemented in time. The study of the COVID-19 outbreak pattern of China may help in controlling the disease in other countries. As per the pattern of China, it may be understood that the effect of this disease in India will be at peak during the third and fourth weeks of April 2020. It is essential to discuss that these predictions are made on the basis of a comprehensive study of China. The strategies to reduce/control COVID-19 cases include a national lockdown of 21 days, public transport control, specialized medical support for affected populations, and others. If these measures are strictly followed, then results may be different.

Our estimate shows that the mortality rate in India is predicted to be around 2.39%. Globally, a 3.4% mortality rate is estimated by the WHO as of March 3, 2020.^9^ This mortality rate may vary in different states of India. It is important to emphasize that the predicted mortality rate could be different from the actual mortality rate looking at the measures taken by the Government of India.

This study has not taken into consideration several other socioeconomic factors that may affect the spreading of COVID-19. These factors may be education, economic conditions, medical facilities, climatic conditions, religious beliefs, and several others.

## CONCLUSION

The COVID-19 pandemic may be controlled if sufficient measures are taken to control the disease. The prediction about the pattern of the outbreak in India may help policy-makers take a comprehensive and necessary action. The number of deaths may be controlled as compared to China because of the precautionary measures that have already been taken in the country. The further study of other factors like education, economic conditions, medical facilities, climatic conditions, religious beliefs, and so forth may strengthen the prediction and help in controlling the outbreak of COVID-19. Also, this study may further be extended for predicting the outbreak in other countries.
